# NAT2 Acetylation Status Predicts Hepatotoxicity During Antituberculosis Therapy: Cumulative Risk Analysis of a Multiethnic Cohort

**DOI:** 10.3390/ijms26083881

**Published:** 2025-04-19

**Authors:** Marco Schiuma, Sofia Dinegro, Vera Battini, Alessandro Torre, Alice Covizzi, Aurora Civati, Miriam Galimberti, Ilaria Mariani, Giulia Mosini, Carla Carnovale, Agostino Riva, Andrea Gori, Spinello Antinori, Emilio Clementi, Sonia Radice, Stefania Cheli

**Affiliations:** 1 Department of Infectious Disease, ASST Fatebenefratelli Sacco, Luigi Sacco Hospital, 20157 Milan, Italy; schiuma.marco@asst-fbf-sacco.it (M.S.); torre.alessandro@asst-fbf-sacco.it (A.T.); covizzi.alice@asst-fbf-sacco.it (A.C.); agostino.riva@unimi.it (A.R.); andrea.gori@unimi.it (A.G.); spinello.antinori@unimi.it (S.A.); 2 ICPS, Pharmacovigilance & Clinical Research, Department of Biomedical and Clinical Sciences, ASST Fatebenefratelli Sacco, Luigi Sacco Hospital, Università degli Studi di Milano, Via G. B. Grassi 74, 20157 Milan, Italy; sofia.dinegro@studenti.unimi.it (S.D.); ilaria.mariani@unimi.it (I.M.); mosini.giulia@asst-fbf-sacco.it (G.M.); carla.carnovale@unimi.it (C.C.); emilio.clementi@unimi.it (E.C.); sonia.radice@unimi.it (S.R.); 3 Department of Biomedical and Clinical Sciences, Università degli Studi di Milano, 20122 Milan, Italy; civati.aurora@asst-fbf-sacco.it (A.C.); galimberti.miriam@asst-fbf-sacco.it (M.G.); 4 Scientific Institute IRCCS Eugenio Medea, 23842 Bosisio Parini, Italy

**Keywords:** tuberculosis, antituberculosis drugs, hepatotoxicity, genetic polymorphism, NAT2 enzyme, acetylation phenotype

## Abstract

Antituberculosis drug-induced hepatotoxicity (ATDH) is a common adverse drug reaction often requiring treatment interruption, complicating tuberculosis management. The slow acetylator phenotype, characterized by reduced N-acetyltransferase 2 (NAT2) enzyme activity, is associated with increased hepatotoxicity risk, while rapid acetylators are associated with a higher risk of therapeutic failure. This study investigates the association between the NAT2 acetylation phenotype and ATDH occurrence, with an emphasis on its predictive value in regard to a multiethnic population and its impact on the timing of ATDH onset. A retrospective observational study was conducted on tuberculosis patients treated at Luigi Sacco Hospital, Milan, Italy (July 2020–September 2023). The *NAT2* genotyping identified slow and rapid/intermediate acetylators. Cumulative incidence analysis and Fine–Gray competing risks regression models were used to assess ATDH risk and onset timing. Among 102 patients, 21.6% developed ATDH, including 16.7% with slow and 4.9% with rapid/intermediate acetylators. ATDH onset was significantly earlier in regard to slow acetylators (median 0.5 vs. 2 months, interquartile range-IQR: 0.5–3 vs. 1.7–5.5). Slow acetylators were associated with a higher risk of developing ATDH (Sub-distribution hazard ratio, SHR = 3.05; 95% confidence interval-CI: 1.17–7.95; *p* = 0.02), even after adjusting for confounders. The NAT2 acetylation phenotype strongly influences ATDH risk and timing. Early acetylator status identification may enable dose adjustments, enhancing treatment safety. These findings highlight the role of pharmacogenetics in optimizing antituberculosis therapy by improving efficacy and minimizing toxicity.

## 1. Introduction

Antituberculosis drug-induced hepatotoxicity (ATDH) remains a significant clinical challenge, contributing to treatment failure and increased mortality in tuberculosis (TB) patients [1,2,3]. The standard six-month TB regimen includes an initial two-month phase involving rifampin (RIF), isoniazid (INH), pyrazinamide (PZA), and ethambutol (EMB), followed by a four-month continuation phase involving INH and RIF [4,5]. The risk of developing hepatotoxicity during this treatment varies significantly among individuals, ranging from 2 to 28%, highlighting the prominence of individual susceptibility [1,6,7]. Emerging [8,9,10] evidence indicates that genetic variations in drug-metabolizing enzymes (DMEs) significantly influence interindividual variability in regard to anti-TB drug metabolism, thereby affecting patients’ susceptibility to hepatotoxicity [11,12,13,14]. Among the first-line TB drugs, INH is the leading cause of hepatotoxicity, followed by PZA and RIF [15,16]. Acetylhydrazine, a key INH metabolite produced by the enzyme N-acetyltransferase 2 (NAT2), is considered a major contributor to INH-induced hepatotoxicity [13,17]. Consequently, the NAT2 gene, which encodes this enzyme, has emerged as a key focus in studies investigating the genetic predisposition to ATDH [18]. The activity of NAT2 is genetically determined and primarily depends on the number of active alleles [18,19]. Studies suggest that genetic polymorphisms are associated with interindividual variability in terms of both the toxicity and efficacy of INH [13,20,21]. Slow acetylators, with reduced NAT2 enzyme activity, metabolize INH more slowly, with increased exposure to and a higher risk of developing ATDH during the application of the standard regimen. Conversely, rapid acetylators, with two active alleles, are associated with a greater risk of treatment failure, likely due to insufficient INH exposure [18,20,21]. These findings suggest that the current internationally recommended INH dosage is excessive for slow acetylators and insufficient for rapid acetylators, highlighting the potential role of a pharmacogenetic-guided dosage to reduce adverse outcomes and improve efficacy. With respect to the timing of adverse outcomes, the relevant guidelines and studies suggest the need for regular liver function monitoring to prevent or mitigate ATDH, but there is no consensus on the optimal timing for such monitoring [22,23,24], possibly because of a lack of evidence on the acetylation status of TB patients and the timing of ATDH onset. In our previous study [25], we provided evidence that the NAT2 acetylation status is significantly associated with the development of ATDH in a multiethnic cohort receiving standard anti-TB therapy. In particular, we demonstrated that slow acetylators are associated with a significantly higher risk of developing ATDH compared to rapid/intermediate acetylators. In the present study, we extend this previous work by investigating the timing of ATDH onset in relation to the patient’s acetylation status, using the same multiethnic cohort. Our aim is to explore whether the NAT2 genotype can be used to predict not only the risk, but also the timing of ATDH onset, thereby improving the early identification of patients at risk and supporting genotype-guided treatment adjustments, thereby optimizing both the safety and efficacy of anti-TB therapy. To our knowledge, this is the first study to address these relationships through the use of temporal analysis and provides new insights into the role of the NAT2 genotype in preventing ATDH and optimizing treatment monitoring.

## 2. Results

### 2.1. Cohort Characteristics

The demographic and clinical characteristics of the included patients (n = 102) are presented in Table 1. The median age was 44 years (IQR: 34–55), with 80.4% of patients under 60 years old. The slow group comprised of 78.6% individuals below 60 years old, compared to 82.6% of the rapid/intermediate group. Males constituted 46.1% of the cohort, evenly distributed in regard to slow acetylators (50%), but less prevalent in regard to rapid/intermediate acetylators (41.3%). The median BMI was 22.5 kg/m^2^ (IQR: 19.6–26.9), with 85.3% of patients having a BMI ≥ 18.5 kg/m^2^. Ethnic representation varied across the groups, with European (30.4%) and African (20.6%) being the predominant categories. TB localization showed a nearly equal distribution of pulmonary (40.9%) and non-pulmonary cases (39.8%), with 19.3% of patients presenting with both. The subgroup analysis indicated minor variations in the localization distribution between slow and rapid/intermediate acetylators.

### 2.2. Distribution of NAT2 Genotypes and ATDH

The distribution of the NAT2 genotype frequencies, phenotypic profiles, and their association with ATDH are presented in Table 2. Among the 102 patients, 56 (54.9%) were classified as slow acetylators, while 46 (45.1%) were classified as rapid/intermediate acetylators. In the slow group, the most common genotypes were NAT2*5/*6 (14.7%), NAT2*5/*5 (13.7%), and NAT2*6/*6 (8.8%). Notably, 17 (77.3%) of the patients who developed ATDH (first event) belonged to the slow group, indicating a significant association between this phenotype and hepatotoxicity risk (*p* = 0.03). Conversely, in the rapid/intermediate group, the most frequent genotypes were NAT2*1/*6 (16.7%), NAT2*1/*5 (14.7%), and NAT2*1/*1 (8.8%). Only five (22.7%) of the patients who developed ATDH were from this group, suggesting a lower prevalence of hepatotoxicity compared to the slow acetylator group. In particular, the NAT2*1/*1 genotype, representing the rapid acetylator phenotype, was not associated with any cases of hepatotoxicity, further supporting the protective role of this genotype against ATDH.

### 2.3. Acetylation Status and Treatment-Related Events

**Figure 1:**
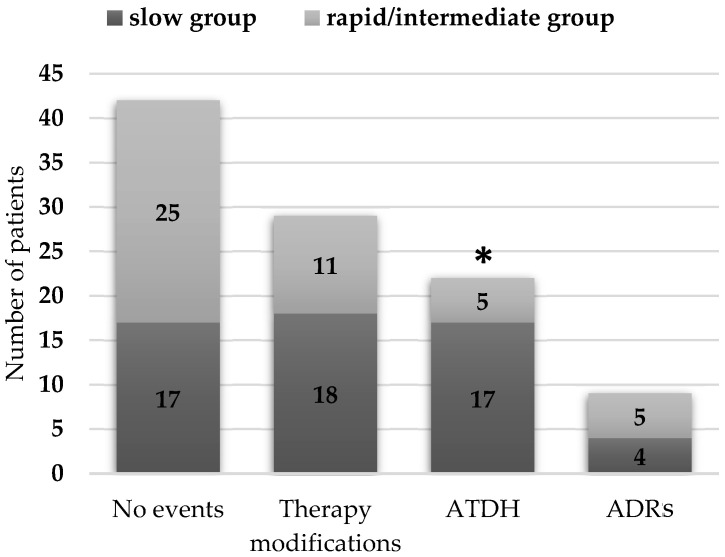
Distribution of acetylation status in relation to treatment-related events. The asterisk (*) indicates a statistically significant difference between the two groups, as determined by a chi-square test (χ^2^ = 8.101, *p* = 0.044).

Out of the 102 individuals in the cohort, 22 (21.6%) developed ATDH as a first event, of which 17 (16.7%) were in the slow group and 5 (4.9%) were in the rapid/intermediate group. Other ADRs, primarily dermatological manifestations, were observed in nine patients (8.8%), including four slow acetylators and five rapid/intermediate acetylators. Notably, among these nine patients, five individuals (three slow acetylators and two rapid/intermediate acetylators) experienced at least two ADRs. Twenty-nine patients (28.4%) from the total cohort required treatment modifications to their initial standard therapy, primarily due to drug-sensitive TB, resulting in the discontinuation of EMB from the regimen (14.7%), the optimization of therapy with the addition of a fluoroquinolone (4.9%), or INH resistance (4.9%). Among the patients who underwent treatment modifications, 18 (17.6%) were in the slow group, while 11 (10.8%) were in the rapid/intermediate group. As illustrated in Figure 1, significant differences were observed in the distribution of the acetylation status with respect to the occurrence of treatment-related events. The most striking disparity was noted in regard to the incidence of ATDH, which was significantly higher in the slow acetylator group.

### 2.4. Cumulative Incidence of ATDH

**Figure 2:**
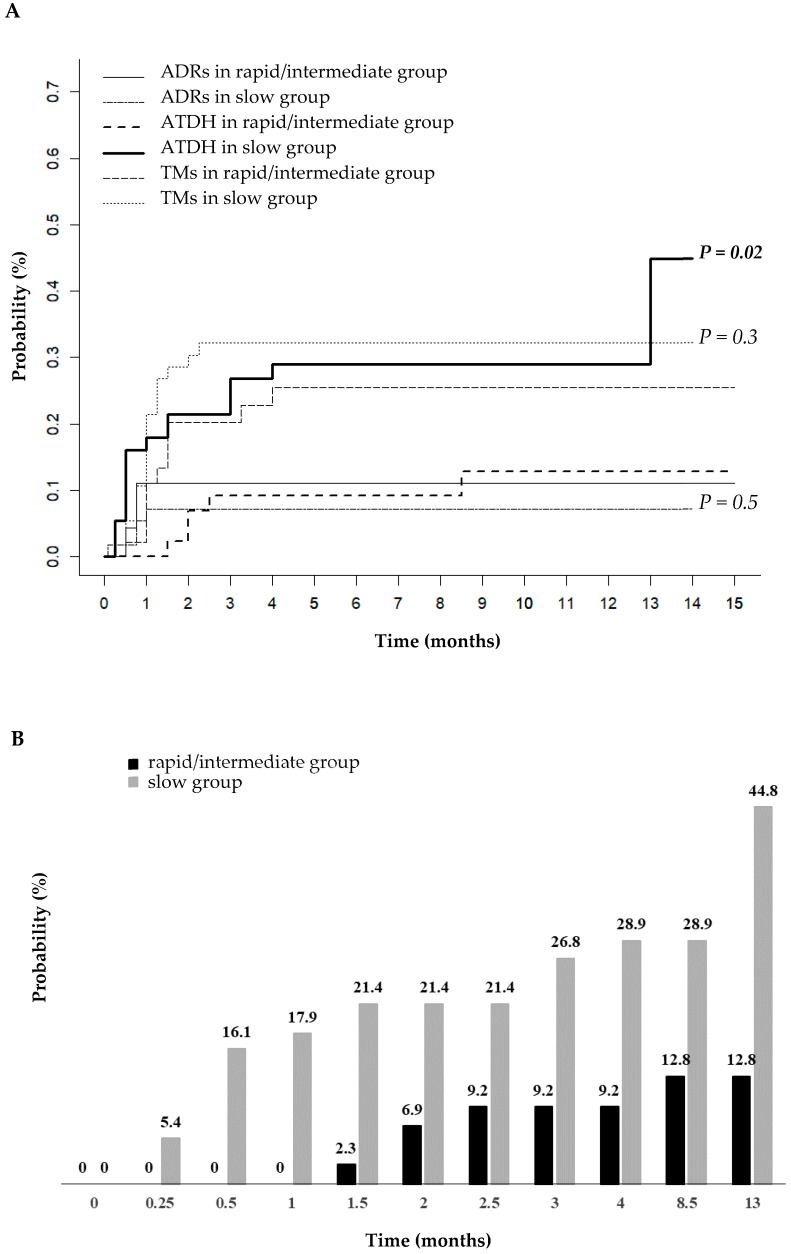
(**A**) Cumulative incidence curves for NAT2 acetylation status related to the development of ATDH, other adverse events, and treatment modifications. (**B**) Cumulative incidence of ATDH based on patient’s acetylation status over time. P: the *p*-value indicates the difference between the slow group and the rapid/intermediate group for each specific variable (Gray’s test). Abbreviations: ADRs = adverse drug reactions; ATDH = antituberculosis drug-induced hepatotoxicity; TMs = treatment modifications.

The probability of developing ATDH over time was analyzed in relation to the patient’s acetylation status, accounting for other adverse drug reactions (ADRs) or treatment modifications. Cumulative incidence analysis revealed a difference in the probability of developing ATDH over time between the slow acetylator and rapid/intermediate acetylator group (Gray’s test, *p* = 0.02) (Figure 2A). No differences were observed among the acetylation phenotypes concerning the incidence of other ADRs (*p* = 0.5) or the need for treatment modifications (*p* = 0.3). The cumulative incidence analysis also showed that ATDH occurred earlier in the slow group, with a median onset time of 0.5 (IQR: 0.5–3) months, compared to 2 (IQR: 1.7–5.5) months in the rapid/intermediate group. Within one week of starting anti-TB therapy, the slow acetylator group had a 5.4% probability of developing ATDH, which tripled to 16.1% by the second week. In contrast, the rapid/intermediate acetylator group maintained a probability of zero up to 1.5 months, which then gradually increased to 2.3% and reached 12.8% at 13 months. Over the same period, the slow acetylator group showed a markedly higher probability of developing ATDH of 44.8% (Figure 2B). A Fine and Gray model confirmed a higher risk of ATDH among the slow acetylator group compared to the rapid/intermediate acetylator group, with a sub-distribution hazard ratio (SHR) of 3.05 (95% CI: 1.17–7.95; *p* = 0.02). This association remained significant after the adjustment for potential confounders, including age, BMI, sex, alcohol consumption, concurrent viral infections, infection localization, and concomitant drugs (adjusted SHR = 2.56; 95% CI: 1.03–6.41; *p* = 0.04).

## 3. Discussion

This study estimated the cumulative risk of developing ATDH among slow and rapid/intermediate acetylator patient groups, accounting for competing events, such as the occurrence of ADRs or possible treatment modifications. The slow acetylator group exhibited a shorter time to ATDH onset compared to the rapid and intermediate acetylator group. The slow acetylator group also exhibited a 3-fold increased risk of developing ATDH during therapy, even after accounting for other factors influencing the probability of ATDH occurrence. These results are consistent with previous studies conducted on specific ethnic groups [26,27]; however, to our knowledge, no prior research has been conducted on a multiethnic cohort like ours [25]. Notably, within the first week of anti-TB therapy, the slow acetylator group presented a 5% probability of developing ATDH, which tripled to around 16% by the second week. In contrast, the rapid and intermediate acetylator group did not experience ATDH until 1.5 months into treatment, with a cumulative probability of 2%, rising gradually to around 13% by 13 months. In the same period, the risk increased to 45% in the slow acetylator group.

These findings emphasize the importance of identifying slow acetylator patients early given their significantly higher and earlier risk of hepatotoxicity, particularly during the intensive phase of therapy (1.5 months), where the probability of developing ATDH for the slow acetylator group was 21.4%. Frequent follow-ups during this period are essential to detect early signs of hepatotoxicity and implement timely interventions. These results also explain a previous observation by our group involving the same cohort, where we found that NAT2 slow acetylators are associated with an elevated risk of hepatotoxicity [25].

Limited data are available in the literature regarding the relationship between the acetylation status of the NAT2 enzyme and the timing in regard to the onset of ATDH. A recent study from China showed an earlier onset of liver damage compared to the overall mean time in patients with slow acetylation genotypes, while those with a rapid acetylation genotype showed a shorter recovery time for their liver function [28]. These differences were not statistically significant (*p* > 0.05), as were those of another report [11]; our analysis confirms these initial evaluations and extends their impact.

A pharmacogenetic clinical trial described a mean time to ATDH onset of 5.4 months among patients with slow acetylators receiving standard therapy [20]. In particular, this study highlights how the NAT2 genotype-guided approach reduced the overall incidence of unfavorable events, both hepatotoxicity and early treatment failure, compared to the conventional regimen, within a Japanese cohort. The Asian population differs significantly from the multiethnic cohort in our study, where patients with slow acetylators are more common and are at greater risk of developing ATDH, unlike the predominantly Japanese group where patients with rapid acetylators are more prevalent. Other studies have focused on the timeline of hepatotoxicity development, with a study conducted in India reporting that ATDH occurred most frequently within the first 15 days of anti-TB treatment, accounting for a cumulative total of 76.8% of cases within the first two months of treatment [29]. A cohort study conducted in southern Ethiopia found that the onset of ATDH typically occurred between 13 and 58 days after treatment initiation, with a median onset of 26 days and an incidence of 8%, among 124 patients studied [30]. The primary risk factor associated with the development of ATDH in that study was chronic high alcohol intake. In a prospective observational study evaluating risk factors for ATDH in a Nepalese patient population, the onset of hepatotoxicity occurred between 12 and 60 days, with a median onset time of 28 days and an incidence of around 8% [31]. None of these studies had a sufficient sample size to properly examine the timeline of hepatotoxicity development nor did they investigate the pharmacogenetic phenotypes. Our study identified 22 (21.6%) cases of ATDH and 80 cases (78.4%) that did not develop it. This sample size was such that it allowed meaningful comparisons, particularly concerning the NAT2 acetylation status, a factor that previous studies did not explore. By integrating the patient’s acetylator status into the analysis, our study provides novel insights into the genetic factors influencing hepatotoxicity development. This approach improves our understanding of risk stratification and emphasizes the role of pharmacogenetics in optimizing the safety of patients during TB therapy. These findings also highlight the importance of personalized medicine in tailoring the treatment to reduce adverse effects, which has been overlooked in earlier studies with smaller sample sizes or limited focus on the acetylation status. Our data suggests that monitoring should begin as early as the first two weeks of therapy, during which initial signs of toxicity may appear. They also suggest the importance of frequent follow-ups throughout the first 46 days, a high-risk period where ATDH incidence increases significantly, as supported by previous studies that similarly observed a higher concentration of hepatotoxicity events within the early weeks of anti-TB treatment [32,33]. The patients with slow acetylators in our cohort, in fact, exhibited a higher probability (21.4%) of developing hepatotoxicity during this timeframe, highlighting the need for a personalized approach to managing patients with this phenotype. Screening for NAT2 polymorphisms before initiating therapy would allow for dose adjustments of INH, optimizing anti-TB treatment, while minimizing the risk of severe adverse reactions like ATDH. Our study is, to the best of our knowledge, the first to highlight the influence of the patient’s NAT2 acetylation status on the timing of ATDH onset in a multiethnic population characterized by a high prevalence of slow acetylator phenotypes. Our findings may imply that the standard dosing of INH for patients with slow acetylators may be excessively high, resulting in prolonged exposure to its intermediate metabolite, hydrazine. Hydrazine is highly reactive and can lead to oxidative stress, mitochondrial dysfunction, and the production of reactive oxygen species (ROS), contributing to hepatocyte damage [34,35]. The combination of toxic metabolite accumulation, oxidative stress, and immune responses may trigger a hepatotoxic cascade [36,37]. These results also suggest the need for dose adjustments based on the patient’s NAT2 acetylation status, particularly for patients with slow acetylators, to mitigate the risk of hepatotoxicity.

This study has limitations. A larger sample size would be needed to increase the statistical power and allow for more generalizable results. The study did not evaluate the potential roles of other genetic factors or the effects of individual medications on hepatotoxicity. Another limitation of this study is the use of a dominant model to classify different acetylation phenotypes, which grouped intermediate and rapid acetylators together. While this approach was chosen due to the low prevalence of patients with rapid acetylators and their functional similarity to patients with intermediate acetylators, it may overlook potential differences in drug metabolism and toxicity risk between these two subgroups. Additionally, this classification does not account for other genetic and non-genetic factors that may influence NAT2 enzymatic activity, such as liver function, concomitant medications, or environmental exposure. These factors could contribute to interindividual variability in drug responses, which may not be fully captured by the acetylation phenotype classification used in this study. Future research should explore alternative classification models or integrate additional covariates to improve the predictive accuracy of NAT2-based stratification.

Despite these limitations, this study, based on retrospective analysis of real-world data, provides valuable insights into the safety and tolerability of standard anti-TB treatment regimens.

## 4. Materials and Methods

### 4.1. Study Design and Population

This observational cohort study utilized data that were retrospectively collected from patients with TB disease or latent TB infection (TBI) enrolled with the Department of Infectious Diseases at Luigi Sacco Hospital in Milan, Italy, between July 2020 and September 2023. All the participants, some of whom had previously been admitted to different hospitals in the Lombardy Region, took part in follow-ups as outpatients at the Luigi Sacco Hospital Tuberculosis Clinic. All the methodological details, including the inclusion and exclusion criteria, treatment protocols, and data collection procedures were conducted as previously described in detail [25]. In summary, the included patients were adults (≥18 years) who received standard initial therapy for active TB, comprising INH (5 mg/kg), RIF (10 mg/kg), PZA (15–30 mg/kg), and EMB (15–20 mg/kg), or treatment for latent TBI with anti-TB drugs, including INH combined with RIF. Eligible participants had normal serum alanine aminotransferase (ALT) and bilirubin levels, no symptoms of liver dysfunction prior to treatment, and provided informed consent. The exclusion criteria comprised liver dysfunction (including biliary causes), prior anti-TB therapy, an anti-TB regimen not including INH or for drug-resistant TB, pregnancy or lactation, concurrent use of hepatotoxic drugs, abnormal hepatic function based on baseline testing, resistance to INH at treatment initiation, or refusal to provide informed consent. The patient’s complete medical history and clinical assessments were obtained from the start of the therapy until the end of the follow-up period.

For the genotype analysis, six NAT2 polymorphisms were identified based on the core sequence variants defined by the PharmVar and PharmGKB databases: NAT2*5 c.341 T>C rs1801280; NAT2*6 c.590 G>A rs1799930; NAT2*7 c.857 G>A rs1799931; NAT2*11 c.481 C>T rs1799929; NAT2*13 c.282 C>T rs1041983; and NAT2*14 c.191 G>A rs1801279. All the genotypes were determined by real-time PCR, using LightSNiP assays (TIB-MolBiol, Berlin, Germany) on a LightCycler 480 (Roche Diagnostics, Basel, Switzerland), according to the manufacturer’s instructions.

### 4.2. Acetylation Phenotype Prediction

The NAT2 allele definitions in this study were based on the legacy nomenclature in the PharmVar database (https://www.pharmvar.org/gene/NAT2, accessed on 29 October 2024). The wild-type NAT2*1 allele, encoding high enzymatic activity, was used as the reference. Alleles NAT2*11 and NAT2*13 were considered functionally equivalent to NAT2*1, conferring a rapid acetylator phenotype. In contrast, alleles NAT2*5, NAT2*6, NAT2*7, and NAT2*14 were associated with reduced enzymatic activity and classified as slow acetylator alleles. The acetylation phenotype was inferred based on the combination of detected alleles: individuals homozygous for slow alleles were classified as slow acetylators; individuals homozygous for rapid alleles were classified as rapid acetylators; heterozygous individuals carrying one slow and one rapid allele were categorized as intermediate acetylators. For individuals who were double heterozygous for slow alleles, haplotype inference was performed considering linkage disequilibrium (LD) estimates among the SNPs and previously reported haplotype frequencies. In such cases, the presence of NAT2*11 and NAT2*13 was evaluated to refine the diplotype classification. The accuracy of this prediction approach has been validated in previous studies by correlating NAT2 genotype-derived phenotypes with isoniazid (INH) clearance rates, demonstrating a strong concordance [38,39].

The patients were initially categorized according to three acetylation phenotypes, but were subsequently grouped into two cohorts based on a dominant model, given the low prevalence of rapid acetylators and their functional similarity to intermediate acetylators. Thus, individuals with a slow acetylator phenotype were assigned to the slow group, while those with either an intermediate or rapid acetylator phenotype were classified into the intermediate/rapid group. Patients were prospectively followed from the start of therapy (baseline) until the occurrence of the outcome (i.e., ATDH) or another adverse event, treatment modification, emigration, completion of therapy, end of follow-up, or death.

### 4.3. Outcome of Interest: ATDH

ATDH was defined as: (1) having serum aspartate transaminase (AST) or alanine transaminase (ALT) levels > 5 times the ULN in patients with the absence of symptoms or with a total bilirubin (BIL) level > 3 times the ULN, or (2) an AST or ALT level > 3 times the ULN and a total BIL level > 2 times the ULN in patients with symptoms compatible with hepatitis.

### 4.4. Ethical Issues

This study followed the principles in the Helsinki Declaration and was approved by the Ethical Committee at IRCSS Ospedale San Raffaele, Milan, Italy (protocol code “TUBILI”, number “CET 106-2024”). Written informed consent was obtained from all the participants.

This research is compliant with the Strengthening the Reporting of Observational Studies in Epidemiology criteria (STROBE, Supplementary File S1). The study was registered on ClinicalTrials.gov with the registration number ID NCT06539455 on 1 August 2024 (protocol TUBILI was uploaded).

### 4.5. Statistical Analysis

Comprehensive descriptive analysis was performed based on all the demographic and clinical variables assessed at therapy initiation. Quantitative variables were summarized as the mean ± standard deviation (SD) or median with the interquartile range (IQR), depending on the data distribution, which was evaluated using the Kolmogorov–Smirnov test. Qualitative variables were presented as frequencies and percentages for each category. Group comparisons were conducted using Student’s *t*-test or the Mann–Whitney test for quantitative variables, and the χ^2^ test for qualitative variables. Correlations between quantitative variables were analyzed using Pearson’s or Kendall’s Tau correlation coefficients. Additionally, all the genotypes were tested in regard to the Hardy–Weinberg equilibrium using the χ^2^ test.

Cumulative incidence analysis, accounting for competing risks, was performed to estimate the probability of ATDH development over time in the slow acetylator group compared to the rapid/intermediate acetylator group (reference group). Gray’s test was applied to assess the differences between the cumulative incidence curves.

The risk of developing ATDH in the slow group compared to the rapid/intermediate group was assessed using the Fine and Gray competing risks regression model. In this model, ATDH was defined as the event of interest and was considered as such only when it occurred before any other events, while competing events included treatment modifications (TMs) and the occurrence of other adverse drug reactions (ADRs). By addressing the competing risk structure, the Fine and Gray model provides a more accurate comparison than standard survival analysis methods, such as the log-rank test, which might ignore competing events. The analysis was adjusted for the patient’s acetylation status, age, BMI, sex, alcohol consumption, concurrent viral infections, infection localization, and concomitant drugs. The results were reported as sub-distribution hazard ratios (SHRs), with their corresponding 95% confidence intervals (95% CI).

In all the analyses, a *p* value < 0.05 was considered statistically significant. The statistical analyses were performed using RStudio (The R Foundation for Statistical Computing, version 4.3.3) (www.R-project.org).

## 5. Conclusions

Pharmacogenetic testing of the NAT2 gene was conducted as part of routine clinical practice, underscoring the importance of assessing patients’ acetylation status before starting anti-TB treatment. In the absence of such testing, more frequent monitoring is recommended to proactively manage and prevent early hepatotoxicity. Future studies ought to integrate personalized isoniazid dosing based on both pharmacogenetic and pharmacokinetic data to determine whether an individualized dosing approach can reduce toxicity and improve therapeutic outcomes. Such an approach may not only optimize treatment efficacy, but also reduce the risk of adverse events, such as hepatotoxicity, that represents a significant barrier to successful TB therapy.

## Figures and Tables

**Table 1:** Baseline characteristics of the study population.

Characteristics	Total n (%)	Slow Acetylator n (%)	Rapid/Intermediate Acetylator n (%)	*p*-Value *
Number of patients	102	56 (54.9)	46 (45.1)	
Age, yrs				
Median, (IQR)	44 (34–55)			
<60	82 (80.4)	44 (78.6)	38 (82.6)	
≥60	20 (19.6)	12 (21.4)	8 (17.4)	0.79
Sex				
Males	47 (46.1)	28 (50)	19 (41.3)	
Females	55 (53.9)	28 (50)	27 (58.7)	0.5
BMI (kg/m^2^)				
Median (IQR)	22.5 (19.6–26.9)			
<18.5	15 (14.7)	10 (17.9)	5 (10.9)	
≥18.5	87 (85.3)	46 (82.1)	41 (89.1)	0.48
Ethnicity				
European	31 (30.4)	18 (32.1)	13 (28.3)	
African	21 (20.6)	13 (23.2)	8 (17.4)	
Latin	18 (17.6)	9 (16.1)	9 (19.6)	
Asian	18 (17.6)	8 (14.3)	10 (21.7)	
Indian	14 (13.8)	8 (14.3)	6 (13.0)	0.82
Tuberculosis localization in active tuberculosis (n = 88)				
Pulmonary	36 (40.9)	21 (43.8)	15 (37.5)	
Non-pulmonary	35 (39.8)	18 (37.5)	17 (42.5)	
Both	17 (19.3)	9 (18.7)	8 (20.0)	0.83

Percentages in regard to all the categories refer to the percentages within each column rather than the overall total. * Chi-square statistic test. Abbreviations: BMI = body mass index; IQR = interquartile range.

**Table 2:** Distribution of NAT2 genotype and ATDH.

Acetylator Status	n (%)	n (%)	n (%)	*p*-Value ^#^
	Overall	ATDH	No ATDH	0.03
**Slow**	56 (54.9)	17 (77.3)	39 (48.8)	
NAT2*5/*5	14 (13.7)	5 (22.7)	9 (11.3)	
NAT2*5/*6	15 (14.7)	5 (22.7)	10 (12.5)	
NAT2*5/*7	7 (6.9)	2 (9.1)	5 (6.3)	
NAT2*6/*6	9 (8.8)	3 (13.6)	6 (7.5)	
NAT2*6/*7	8 (7.8)	2 (9.1)	6 (7.5)	
NAT2*6/*14	1 (1.0)	0 (0.0)	1 (1.3)	
NAT2*7/*7	2 (2.0)	0 (0.0)	2 (2.5)	
**Rapid/Intermediate**	46 (45.1)	5 (22.7)	41 (51.2)	
NAT2*1/*1	9 (8.8)	0 (0.0)	9 (11.3)	
NAT2*1/*5	15 (14.7)	2 (9.1)	13 (16.3)	
NAT2*1/*6	17 (16.7)	1 (4.5)	16 (20.0)	
NAT2*1/*7	5 (4.9)	2 (9.1)	3 (3.8)	
**Total** *	102 (100)	22 (100)	75 (100)	

* The total refers to the sum of values within each column; ^#^ *p*-value as a result of chi-square analysis. Abbreviations: n = number of patients; % = frequency of patients; ATDH: antituberculosis drug-induced hepatotoxicity.

## Data Availability

The study was registered on ClinicalTrials.gov with the registration number ID NCT06539455 on 1 August 2024 (protocol TUBILI was uploaded).

## Supplementary Materials

The following supporting information can be downloaded at: https://www.mdpi.com/article/10.3390/ijms26083881/s1.

## Abbreviations

The following abbreviations are used in this manuscript:

ADR	Adverse drug reaction
ALT	Alanine transaminase
AST	Aspartate transaminase
ATDH	Antituberculosis drug-induced hepatotoxicity
BIL	Bilirubin
BMI	Body mass index
CI	Confidence interval
DMEs	Drug-metabolizing enzymes
EMB	Ethambutol
INH	Isoniazid
IQR	Interquartile range
NAT2	N-Acetyltransferase 2
PZA	Pyrazinamide
RIF	Rifampin
SHR	Sub-distribution hazard ratio
TB	Tuberculosis
ULN	Upper limit of normal
