# Supplementary material for: NAT2 Acetylation Status Predicts Hepatotoxicity During Antituberculosis Therapy: Cumulative Risk Analysis of a Multiethnic Cohort
Source: Int J Mol Sci. 2025 Apr 19;26(8):3881. doi: 10.3390/ijms26083881 (PMC12027989; doi:10.3390/ijms26083881)
Supplement: Supplementary file 1 [file ijms-26-03881-s001.zip › ijms-3550964-supplementary.pdf]

Supplementary File S1. STROBE Statement— Checklist of items that were included in: "NAT2 Acetylation Status Predicts Hepatotoxicity During Antituberculosis Therapy: Cumulative Risk Analysis of a Multiethnic Cohort "

	Page	Note
**Title**	**1**	
**Abstract**	**1**	
**Introduction**		
Background/rationale	**2**	*1. Introduction* paragraph
Objectives	**2**	*Introduction*, lines 80-86: "In the present study, we extend this previous work by investigating the timing of ATDH onset in relation to the patient's acetylation status, using the same multiethnic cohort. Our aim is to explore whether the NAT2 genotype can be used to predict not only the risk, but also the timing of ATDH onset, thereby improving the early identification of patients at risk and supporting genotype-guided treatment adjustments, thereby optimizing both the safety and efficacy of anti-TB therapy".
**Methods**		
Study design	**9**	*4.1 Study design and population* paragraph
Setting	**9**	Described in paragraph: *4.1 Study design and population*
Participants	**9**	Described In paragraph: *4.1 Study design and population*
Variables	**9-10 ***	Described in paragraphs: *4.1 Study design and population and 4.3 Outcome of interest: ATDH*
		Described in protocol "TUBILI," registered with ClinicalTrials.gov on August 1, 2024. The study number is NCT06539455
Data sources/ measurement	**9-10 ***	Described in paragraphs: *4.1 Study design and population and 4.3 Outcome of interest: ATDH*
		Described in protocol "TUBILI," registered with ClinicalTrials.gov on August 1, 2024. The study number is NCT06539455
Bias	*****	Described in protocol "TUBILI," registered with ClinicalTrials.gov on August 1, 2024. The study number is NCT06539455
Study size	*****	Described in protocol "TUBILI," registered with ClinicalTrials.gov on August 1, 2024. The study number is NCT06539455
Quantitative variables	**10-11**	Described in paragraph: *4.5 Statistical Analysis*
Statistical methods	**10-11**	*4.5 Statistical Analysis* paragraph
**Results**		
Participants	**2-3**	Described in paragraph: *2.1 Cohort characteristics*
Descriptive data	**2-4**	Described in paragraphs: *2.1 Cohort characteristics and 2.2 Distribution of NAT2 genotypes and ATDH*
		Table 1 and 2
Outcome data	**4-5**	Described in paragraphs: *2.3 Acetylation status and treatment-related events*
		Figure 1
Main results	**5-6**	Described in paragraph: *2.4 Cumulative Incidence of ATDH*
		Figure 2

**Discussion**		
**Key results**	**7**	*Discussion, lines 175-188 "This study estimated the cumulative risk of developing ATDH among the slow, intermediate, and rapid acetylators, accounting for competing events such as the occurrence of ADRs or possible treatment modifications….*
**Limitations**	**8**	*Discussion, lines 255-269:* "This study has limitations…..
**Interpretation**	**7**	*Discussion, lines 243-254:* "*These findings emphasize the importance of identifying slow acetylators…..*
**Generalisation**	**9**	*Discussion, lines 265-269:* "*Our study is, to the best of our knowledge, the first to highlight the influence of NAT2 acetylation status on the timing of ATDH onset in a multiethnic population characterized by a high prevalence of slow acetylator phenotypes.…*

*https://clinicaltrials.gov/
